# DUSP4 is associated with increased resistance against anti-HER2 therapy in breast cancer

**DOI:** 10.18632/oncotarget.20430

**Published:** 2017-08-24

**Authors:** Otília Menyhart, Jan Budczies, Gyöngyi Munkácsy, Francisco J. Esteva, András Szabó, Teresa Puig Miquel, Balázs Győrffy

**Affiliations:** ^1^ Semmelweis University 2nd Department of Pediatrics, Budapest, Hungary; ^2^ Institute of Pathology, Charité University Hospital, Berlin, Germany; ^3^ Clinical Cancer Center, NYU Langone Medical Center, New York, NY, USA; ^4^ New Terapeutics Targets Laboratory (TargetsLab), Department of Medical Sciences, University of Girona, Girona, Spain; ^5^ MTA TTK Lendület Cancer Biomarker Research Group, Institute of Enzymology, Budapest, Hungary

**Keywords:** DUSP4, targeted therapy, trastuzumab, biomarker, breast cancer

## Abstract

The majority of patients develop resistance against suppression of HER2-signaling mediated by trastuzumab in HER2 positive breast cancer (BC). HER2 overexpression activates multiple signaling pathways, including the mitogen-activated protein kinase (MAPK) cascade. MAPK phosphatases (MKPs) are essential regulators of MAPKs and participate in many facets of cellular regulation, including proliferation and apoptosis. We aimed to identify whether differential MKPs are associated with resistance to targeted therapy in patients previously treated with trastuzumab. Using gene chip data of 88 HER2-positive, trastuzumab treated BC patients, candidate MKPs were identified by Receiver Operator Characteristics analysis performed in R. Genes were ranked using their achieved area under the curve (AUC) values and were further restricted to markers significantly associated with worse survival. Functional significance of the two strongest predictive markers was evaluated *in vitro* by gene silencing in HER2 overexpressing, trastuzumab resistant BC cell lines SKTR and JIMT-1. The strongest predictive MKPs were DUSP4/MKP-2 (AUC=0.75, *p*=0.0096) and DUSP6/MKP-3 (AUC=0.77, *p*=5.29E-05). Higher expression for these correlated to worse survival (DUSP4: HR=2.05, *p*=0.009 and DUSP6: HR=2, *p*=0.0015). Silencing of DUSP4 had significant sensitization effects – viability of DUSP4 siRNA transfected, trastuzumab treated cells decreased significantly compared to scramble-siRNA transfected controls (SKTR: *p*=0.016; JIMT-1: *p*=0.016). In contrast, simultaneous treatment with DUSP6 siRNA and trastuzumab did not alter cell proliferation. Our findings suggest that DUSP4 may represent a new potential target to overcome trastuzumab resistance.

## INTRODUCTION

Human epidermal growth factor receptor 2 (HER2) is a critical member of the epidermal growth factor receptor (EGFR) transmembrane receptor tyrosine kinase (RTK) family. HER2 does not possess a known ligand, but its dimerization domain is in continuous open conformation making it the preferred dimerization partner of other EGFR RTKs. The hetero- or homodimerization induces activation of the PI3K/Akt, Ras/MAPK, and JAK/STAT pathways, leading to increased cell proliferation and survival [1]. Targets of the receptor also modulate angiogenesis via activation of VEGFA, suppress apoptosis via NFKB and control the cell cycle via p27, cyclin D1 and D2 [2].

Breast cancer is the leading cause of cancer related deaths in the age group of 45-55 year old women. HER2 is overexpressed in 20-25% of invasive breast cancer [3], that translates to over 46 thousand new HER2-positive cases each year in the USA alone [4]. HER2-positivity confers aggressive tumor growth, high incidence of local recurrence, variable response to conventional chemotherapy and worse prognosis in general [5–7]. The introduction of the anti-HER2 monoclonal antibody trastuzumab in 1998 transformed the course of disease for these patients, and today HER2-positivity confers better prognosis compared to receptor negativity [8]. Trastuzumab is a humanized monoclonal antibody (mAb) that attaches to the fourth extracellular domain of the HER2 receptor suppressing HER2 signaling. Trastuzumab improves objective response rate (ORR), progression free survival (PFS) and overall survival (OS) of HER2-positive patients [9]. It also provides advantage for disease-free survival (DFS) and OS as adjuvant therapy combined with chemotherapy [10, 11], or as monotherapy after chemotherapy [12]. Recently, excellent prognosis was reported in patients with small, stage I, node negative tumor after trastuzumab treatment combined with paclitaxel [13]. Neoadjuvant trastuzumab combined with chemotherapy improved pathologic complete remission (pCR) in phase II and III clinical trials [14].

Anti-HER2 therapy is a targeted therapy as response can be expected in HER2-positive patients only – however, ORR in patients with metastatic disease is not more than 50% [15]. In addition, 70% of HER2 positive patients demonstrate intrinsic or secondary resistance to trastuzumab [16]. The major resistance mechanisms include: **a.** impaired access to the binding site; **b.** augmented signaling through other ERBB family receptors and their ligands; **c.** activation of HER2 targets by alternative heterodimers and initiation of collateral signal transduction; **d.** signaling triggered by downstream pathway members, such as activating mutations in PIK3CA or loss of tumor suppressor PTEN; **e.** altered expression of cell cycle and apoptotic regulators; and **f.** hormone receptor status (for a recent review of resistance mechanisms see [17]). Most of the numerous biomarkers of resistance proposed in pre-clinical studies delivered heterogeneous results when evaluated in clinical trials [18, 19]. The extent of HER2 amplification and protein overexpression predicts response to trastuzumab, and remains currently the single marker utilized for patient selection [18]. To improve response rate, it will be imperative to identify additional biomarkers capable of further differentiating patients regarding their resistance to anti-HER2 treatment.

HER2 overexpression activates multiple signaling pathways, including the mitogen-activated protein kinase (MAPK) cascade. MAPKs, such as ERK, JNK and p38 drive many facets of cellular regulation, including proliferation and differentiation [20]. Constitutive activation of MAPKs has been associated with cancer development [21]. Tumor relevant MAPKs are regulated by mitogen-activated protein kinase phosphatases (MKPs), a subgroup of dual-specificity phosphatases (DUSPs). The ten members of the MKP family are subdivided into three groups based on cellular localization and substrate specificity, and exert complex spatial and temporal control on MAPK signaling [22]. Both up- and down-regulation of MKPs were linked to the development of various human cancers [23]. The role of MKPs is highly tissue specific and also depends on the mutations present in malignant transformations [24]. Dysregulation of MKPs, such as DUSP1/MKP-1, mediates chemoresistance in cancer cells [25–27].

While tyrosine kinases received much attention in HER2 driven tumorigenesis, the role of MPKs remains mostly unexplored. The failure of trastuzumab to suppress HER2-mediated signaling suggest placing regulators of proliferation and apoptosis, such as MKPs, under close scrutiny. Here we hypothesized that differential expression of MKPs is associated with therapy resistance in previously trastuzumab treated patients. In our study candidate MKPs were identified using two independent transcriptomic cohorts, and were restricted to markers associated with survival benefit in HER2-positive patients. The strongest predictive biomarkers, DUSP4/MKP-2 and DUSP6/MKP-3 were evaluated *in vitro*, in which their functional role was investigated in trastuzumab resistant cell lines.

## RESULTS

### Identification of biomarker-candidates in trastuzumab-treated breast cancer cohorts

All the HER2 positive patients included in the statistical analysis received trastuzumab and chemotherapy. In the statistical analysis, only samples for which response data were available were included. Aggregate clinical, pathological and survival characteristics for the included datasets are presented in Table 1.

**Table 1:** Comparison of the microarray datasets used to assess the association of gene to response against anti-HER2 therapy

Dataset	Affymetrix dataset	Agilent dataset	[57]
n	50	11	27
Platform	GPL96	GPL1708	GPL5325
**Clinical characteristics**			
- Mean age (years)	**49.42**	**47.43**	**n.a.**
- Lymph node positive (%)	15 (29.4%)	**n.a.**	**n.a.**
- ER status (negative %)	64%	55%	56%
- PR status (negative %)	74%	64%	70%
- HER2 status (%)	Positive (100%)	Positive (100%)	Positive (100%)
**Treatment characteristics**			
- Anti-HER2 therapy	Trastuzumab (100%)	Trastuzumab (100%)	Trastuzumab (100%)
- Chemotherapy (%)	FEC+T (100%)	AC+T (100%)	D+C (100%)
- Response (%)	48%	45.5%	37%

doxorubicin + cyclophosphamide + taxane=AC+T; docetaxel + capecitabine=DC; FEC+Taxol=FEC+T.

We performed ROC analysis for all MKP genes independently on both platforms. Based on AUC values DUSP4 and DUSP6 were the best performing MKPs (average AUC=0.687 for DUSP4 and 0.728 for DUSP6). DUSP1 (AUC=0.678) and DUSP16 (AUC=0.7667) reached significance only in one platform of the two, therefore were excluded from further analysis. The complete list of all significant AUC values from the two platforms for each MKPs are presented in Table 2.

**Table 2:** The list of the 10 MKPs and their response against anti-HER2-treatment in affymetrix and agilent datasets

Symbol	Gene name	Affymetrix dataset (n=50)			Agilent dataset (n=38)		
		Probe ID	AUC	p Value	ProbeID	AUC	p Value
**DUSP1 / MKP-1**	dual specificity phosphatase 1	201041_s_at	n.s.	n.s.	20676	0,678	0,0323
**DUSP2 / PAC1**	dual specificity phosphatase 2	204794_at	n.s.	n.s.	26030	n.s.	n.s.
**DUSP4 / MKP-2**	**dual specificity phosphatase 4**	**204014_at**	**0,686**	**0,0088**	**23420**	**0,687**	**0,0230**
**DUSP5**	dual specificity phosphatase 5	209457_at	n.s.	n.s.	8917	n.s.	n.s.
**DUSP6 / MKP-3**	**dual specificity phosphatase 6**	**208892_s_at**	**0,770**	**3,08E-05**	**24661**	**0,687**	**1,57E-02**
**DUSP7 / MKPX**	dual specificity phosphatase 7	213848_at	n.s.	n.s.	350	0,629	0,0841
**DUSP8**	dual specificity phosphatase 8	206374_at	n.s.	n.s.	32679	n.s.	n.s.
**DUSP9 / MKP-4**	dual specificity phosphatase 9	205777_at	n.s.	n.s.	11958	n.s.	n.s.
**DUSP10 / MKP-5**	dual specificity phosphatase 10	221563_at	n.a.	n.a.	450	0,638	0,0709
**DUSP16 / MKP-7**	dual specificity phosphatase 16	208891_at	0,7667	5,29E-05	14131	n.s.	n.s.
**HER2**	v-erb-b2 erythroblastic leukemia viral oncogene homolog 2	216836_s_at	0,638	4,90E-02	26639	0,809	9,50E-06

DUSP4 and DUSP6 were the only candidates associated with response to trastuzumab in both cohorts. HER2 is included as a control gene.

### Survival analysis

**Figure 1:**
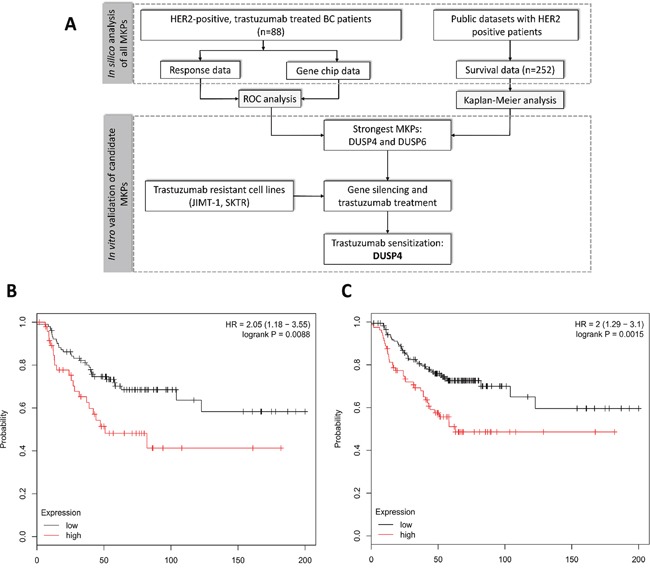
Schematic overview of the study **(A)** and Kaplan-Meier survival plot of the two best performing MKPs, DUSP4 **(B)** and DUSP6 **(C)** in 252 HER2 positive patients.

Survival analysis was drawn from the survival data of 252 HER2 positive patients. All of the patients were HER2 positive (cut off 4800), 65.8% ER positive (cut off 500) both defined by the gene expression. The patients’ clinical characteristics (from the available data) were the following: 51.1 years median age, 2.3 cm average tumor size, 4.7 months median relapse free time, 6.6 months median time to death. The two best performing MKPs, DUSP4 and DUSP6 reached a HR of 2.05 (confidence interval 1.18-3.55, p=0.0088) and 2.0 (1.29-3.1, p=0.0015), respectively. Figure 1B and 1C depict the Kaplan-Meier curves for DUSP4 and DUSP6.

### Expression of HER2 in various BC cell lines

We assessed HER2 expression by qPCR in the breast cancer cell lines SKBr-3, SKTR and JIMT-1. Known as a HER2-overexpresssing cell line, SKBr-3 and its trastuzumab resistant subclone, SKTR, were indeed highly expressing the HER2 mRNA, and expression levels did not differ between parental cells and derived subclones (*p*>0.1). HER2 expression at the mRNA level in SKBr-3 and SKTR cell lines was significantly higher compared to the JIMT-1 cell line (*p*=0.002), even though each of them were designated as HER2-positive.

### Cell proliferation in the presence of trastuzumab

**Figure 2:**
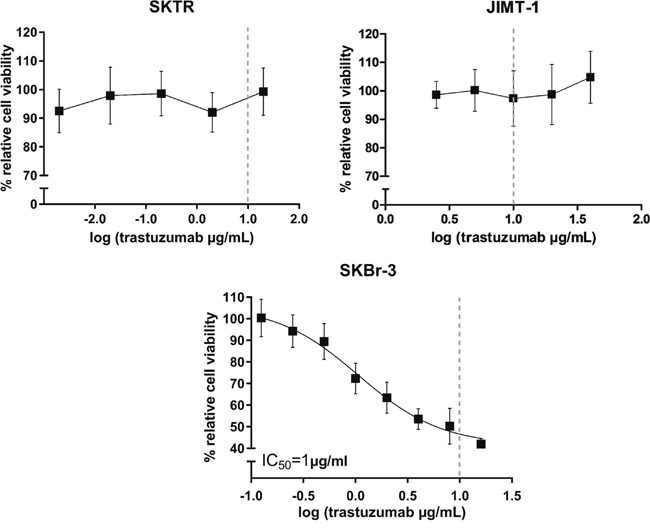
Trastuzumab sensitivity of resistant SKTR and JIMT-1 cell lines Dose-response curves after 72h trastuzumab treatment with concentrations ranging between 0.002-20 μg/mL for SKTR and 2.5-40 μg/mL for JIMT-1 cell lines. % relative cell viability refers to the growth of trastuzumab treated cells relative to untreated control cells. The dashed line represents the pharmacologically relevant concentration of trastuzumab (10 μg/mL). The growth of parental SKBr-3 cell line was inhibited by trastuzumab, with an IC_50_ value of 1μg/mL.

All cell lines were exposed for 72 hours to increasing concentrations of trastuzumab (SKBr-3: 0.125-16 μg/mL; SKTR: 0.002-20 μg/mL; JIMT-1: 2.5-40 μg/mL). In agreement with previous reports, growth of the parental SKBr-3 cell line was inhibited by trastuzumab, with an IC_50_ value of 1μg/mL (Figure 2). Trastuzumab did not inhibit proliferation of SKTR and JIMT1 cells even at the highest concentrations, corresponding approximately to 2 times (SKTR) or 4 times (JIMT-1) of the pharmacologically applicable dose (Figure 2).

### RNA interference decreasing DUSP6 and DUSP4 expression

**Figure 3:**
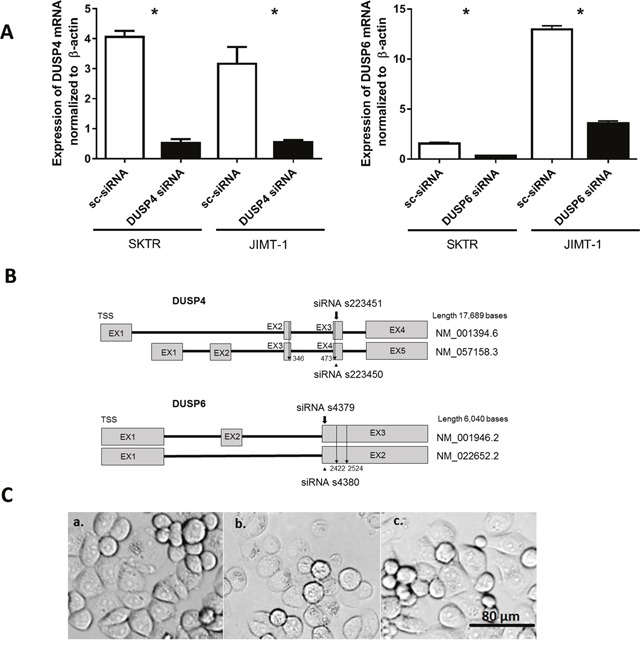
Silencing efficacy after siRNA treatment **(A)** Expression of DUSP4 mRNA andDUSP6 mRNAnormalized to β-actin in Negative control siRNA (sc-siRNA) and DUSP4 siRNA treated SKTR and JIMT-1 cells. **(B)** Schematic view of DUSP4 and DUSP6 genes. BothDUSP4 andDUSP6 possess two isoforms. The position of tested siRNAs is shown, and the location of the more effectively silencing from the two is marked with a bold arrow. Binding location of gene specific primers spanning both isoforms is indicated with a line arrow. **(C)** Morphology of SKTR a. untreated control cells; b. cells treated with DUSP4 siRNA; c. cell treated with Negative control siRNA.

We verified the ability of DUSP6 and DUSP4 siRNAs to reduce the endogenous mRNA levels in SKTR and JIMT-1 cell lines after a 72 hour long siRNA treatment. The silencing efficacy compared to a negative siRNA transfected control in SKTR cell line were 78.3% for DUSP6 and 86.2% for DUSP4, and in JIMT-1 cell line were 72.3% for DUSP6 and 81.7% for DUSP4. Silencing efficacy is illustrated in Figure 3A and binding of the siRNA oligos in Figure 3B. siRNA treatment did not alter the morphology of the cells (Figure 3C).

### Treatment with DUSP4 specific siRNA induces sensitivity to trastuzumab in SKTR and JIMT-1 cell lines

**Figure 4:**
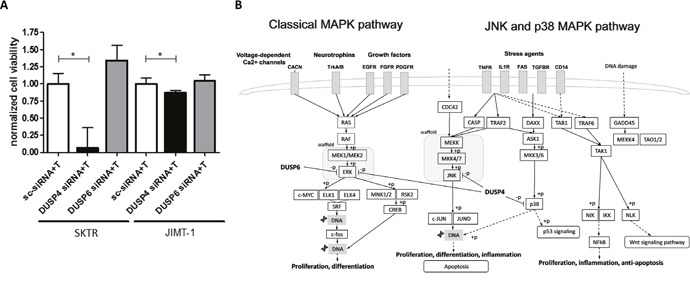
Effects of DUSP4 and DUSP6 silencing on cell viability after trastuzumab treatment **(A)** Normalized viability of SKTR and JIMT-1 cells treated with 10 μg/mL trastuzumab (T) after DUSP4 and DUSP6 silencing compared to Negative control siRNA (sc-siRNA) treated cells (mean with SEM) and **(B)** simplified scheme of the targets of DUSP4 and DUSP6. DUSP4 potentially promotes both survival and apoptosis by dephosphorylating ERK, JNK and p38.

To observe the roles of DUSP6 and DUSP4 in trastuzumab resistance, we combined siRNA transfection with a 10 μg/ml trastuzumab treatment, after which an MTT assay was performed. Viability of transfected SKTR and JIMT-1 cells significantly decreased after DUSP4 siRNA treatment compared to negative control siRNA transfected cells as a consequence of trastuzumab treatment (SKTR: *p*=0.016; JIMT-1: *p*=0.016). In contrast, DUSP6 siRNA transfected and trastuzumab treated cells did not exhibit changes in cell viability (SKTR: *p*>0.1; JIMT-1: *p*>0.1). Results of the trastuzumab combined silencing experiments are illustrated in Figure 4A.

## DISCUSSION

Our goal was to identify biomarkers of resistance for trastuzumab treatment. HER2 overexpression affects growth factor induced signaling, we hypothesized therefore a role of the endogeneous regulators of the mitogen-activated protein kinase (MAPK) cascade, the mitogen-activated protein kinase phosphatases (MKPs). We applied a bottom up approach in the quest for biomarkers. DUSP4/MKP-2 and DUSP6/MKP-3, *in silico* identified candidate MKPs associated with survival, were validated *in vitro* in HER2-overexpressing trastuzumab-resistant cell lines.

In mammalian cells, the MAPK signaling pathway is a key transducer of growth factor signaling that incorporates three family members: the extracellular signal regulated kinases (ERK), the c-Jun N-terminal kinases (JNK), and the p38 MAPKs. Phosphorylation of ERK, JNK and p38 induces transcription factors involved in cell growth. Moreover, depending on the signaling context, JNK and p38 pathways are also implicated in cell death (Figure 4B) [28]. MKPs, as dual-specificity phosphatases dephosphorylate both the phospho-threonine and phospho-tyrosine residues of MAPKs, and provide negative feedback to the MAPK pathways [29]. DUSP4 and DUSP6 belong to different subtypes of MKPs. DUSP6 is based in the cytoplasm and its primary substrate is ERK. DUSP4 is located mainly in the nucleus and can dephosphorylate all three MAPKs [29] (Figure 4B). Different spatial and temporal control of MAPKs and substrate specificity of MKPs may explain the impact of DUSP4 silencing in our study that was not associated with DUSP6 downregulation.

DUSP4 expression has been shown to be altered in a variety of human cancers. Higher expression of DUSP4 was described in primary breast cancer [30], colorectal adenocarcinoma [31], pancreatic cancer [32], human melanoma cells [33], and as a result of chemical hepatocarcinogenesis in rats [34]. DUSP4 overexpression increased proliferation in colorectal carcinoma cell lines [31]. Overall and disease-free survival was associated with DUSP4 expression in colorectal adenocarcinoma [35]. In contrast, activating EGFR-mutation was related to downregulation of DUSP4 in lung adenocarcinomas, and *in vitro* knockdown of DUSP4 increased cell proliferation [36]. DUSP4 is frequently lost in early-onset and high-grade breast carcinomas [37]. In basal-like breast cancers, especially in chemotherapy resistant triple negative breast cancer (TNBC), low DUSP4 mRNA is coupled with high RAS-ERK activation, that also relates to shorter recurrence free survival [38]. Using 230 tissue samples, high DUSP4 mRNA expression was observed in HER2-overexpressing tumors [38]. RNAi-mediated DUSP4 depletion decreased proliferation in mouse mammary tumor cells both *in vitro* and *in vivo* [37, 39]. DUSP4 was identified as one of the genes responsible for resistance against multiple chemotherapy agents including methotrexate, mitoxantrone, mitomycin C and etoposide [40]. In human embryonic kidney cells 293 (HEK293) overexpression of DUSP4 rescued cells from apoptosis when exposed to UV-C or cisplatin treatment by selectively dephosphorylating JNK [41]. In summary, DUSP4 exerts complex control on cell proliferation, and can play opposite role in tumorigenesis depending on tissue and molecular subtype.

Although high expression of both DUSP4 and DUSP6 is associated with worse survival (Figure 1B and 1C), DUSP6 downregulation combined with simultaneous trastuzumab did not reduce cell viability. The role of DUSP6 as a tumor suppressor or potential oncogene is tissue specific. Significant reduction of DUSP6 thwarts negative feedback on ERK, thus increases the activation of ERK1/2 MAP kinase pathway. As a consequence, low DUSP6 expression accelerates cell growth in pancreatic cancer [29]. Conversely, DUSP6 expression was abrogated in invasive pancreatic carcinomas compared to precursor lesions [42]. In contrast, in a model of HER2-positive BC, cells activated with EGF strongly overexpressed DUSP6, while HER2 expression downregulated by a stable intracellular expression of an anti-HER2 antibody resulted in DUSP6 downregulation. Publicly available HER2-positive breast cancer datasets also confirmed DUSP6 overexpression, suggesting a specific involvement of DUSP6 in breast cancer [43]. The potential difference in spatial and temporal control of MAPKs, tissue and substrate specificity of MKPs requires further investigations to explain the lack of impact of DUSP6 downregulation on cell viability.

The simultaneous siRNA and trastuzumab treatment reduced viability in both SKTR and JIMT-1 cell lines. However, comparing the effectiveness of treatment highlights their markedly different responses to DUSP4 siRNA-combined trastuzumab (Figure 4A). SKTR and JIMT-1 cell lines represent different models of HER2-positive BC. HER2 mRNA expression was significantly higher in SKBr-3 and SKTR cells compared to JIMT-1, and similar differences have been described at the protein level [44]. Currently available molecular taxonomy of BC does not distinguish subpopulations within clinically HER2-positive BC patients. However, trastuzumab responsiveness is suggested to be dependent on BC subtypes (e.g. luminal, basal, HER2-enriched and claudin-low) within the clinically HER2-positive BC population [45].

JIMT-1 was derived from a clinically trastuzumab resistant patient [46], and belongs to the basal subtype within the clinically HER2-positive breast cancer populations. JIMT-1 is naturally enriched with stem cell markers, and is distinguished from other HER2 positive cell lines by harboring several co-existing drug resistance mechanisms, including activating mutation of the PIK3CA gene, low PTEN expression, high NRG1 expression, and relatively low expression of HER-2 receptor protein (despite gene amplification) [47]. In contrast, SKBr-3 carries the wild-type of PIK3CA and represents a preclinical model of HER2 gene amplified breast cancer that expresses high levels of EGFR [48]. The different response between SKTR and JIMT-1 to DUSP4 siRNA-combined trastuzumab supports the importance of subdivision of clinically HER2-positive BC populations. However, reversing resistance in both cell lines, that represent different subtypes of HER2-positive BC by silencing a single member of the MAPK pathway, highlights the overarching role of DUSP4 as promising marker of resistance across multiple subtypes. The transient nature of the siRNA treatment did not allow to study long term consequences of DUSP4 knockdown because the effects of RNA silencing started to diminish after 72 hours. To study long term effects of DUSP4 on proliferation would require a different methodology, such as a permanent gene knockdown with a shRNA contruct.

Significant improvement has been achieved in the treatment of HER2-positive breast cancer since trastuzumab became licensed in 1998. However, only a slice of patients enjoy the benefits of anti-HER2 therapy, and inherent or *de novo* resistance poses a serious challenge. In our study we examined the MKP family as potential candidate biomarkers of resistance by using microarray data of trastuzumab treated patients. Applying *in vitro* gene silencing coupled with independent validation in clinical cohorts, we pinpointed *DUSP4* as the most promising MKP correlated to trastuzumab resistance in HER2-positive breast cancer patients. Given the highly tissue specific role of DUSP4, strategies that target DUSP4 could be developed and explored in this patient population.

## MATERIALS AND METHODS

### Trastuzumab-treated breast cancer patients

We used two different discovery cohorts of trastuzumab-treated breast cancer patients: one comprising 50 patients at the MD Anderson Cancer Center by F.J.E., a second dataset of two studies comprising 38 patients published in GEO as GSE22226 and GSE22358 produced using Agilent arrays.

### Processing of microarray data

Microarray data for the 50 patients was generated using Affymetrix HGU133A microarrays following the manufacturer's protocols. The raw array data was MAS5 normalized in the R environment using the affy Bioconductor package [49]. For genes measured by several probe sets, the most reliable probe set was selected using JetSet [50]. Only probe sets reaching a MAS5 expression value of 1000 in at least one of the samples were used in the statistical computations. For GSE22226 and GSE42822 we used the series matrix database containing the normalized gene expression data for all samples. Mapping between different platforms was performed by only using the probes measuring exactly the same sequences. For this, the probe sequences were downloaded from GEO platform database. Only probe sets having at least 20% standard deviation compared to the maximal value were investigated in the analysis (n=13, 295).

### Statistical analyses

ROC analysis was performed in the R statistical environment (http://www.r-project.org) using the ROC Bioconductor library (http://www.bioconductor.org). Statistical significance was set at p<0.05. The two cohorts comprising of 50 and 38 patients were processed separately to avoid batch effects, and only genes resulting in significant correlation in each dataset were considered significant. Mapping between the platforms was performed using the annotation tables of Affymetrix (http://www.affymetrix.com). The final ranking of the genes was performed by computing the average AUC across the two platforms.

### Datasets for survival analysis

GEO (http://www.ncbi.nlm.nih.gov/geo), EGA (http://www.ebi.ac.uk/) and TCGA (http://cancergenome.nih.gov/) were searched using the keywords “breast”, “cancer” and “survival”. All available samples were downloaded and processed as described above. HER2-positivity was determined by using the expression values of the probe set 216836_s_at as described previously [51]. All together 252 HER2 positive patients with available relapse-free survival data were identified. In these, Kaplan-Meier analysis was performed as described previously [52].

### Cell lines and culture

Breast cancer cell lines with an amplified HER2 oncogene were chosen to model HER2-positive trastuzumab resistant breast carcinoma: trastuzumab sensitive SKBr-3, trastuzumab resistant SKTR and JIMT-1. JIMT-1 cell lines were purchased from the American Type Culture Collection (Manassas, VA, USA). JIMT-1 is an inherently trastuzumab resistant estrogen and progesterone negative breast carcinoma cell line from epithelial origin [46]. JIMT-1 cells were cultured in high glucose DMEM (Gibco) supplemented with 10% FBS, penicillin and streptomycin. Cells were housed at 37°C with 5% CO_2_. Cell lines were tested regularly for the presence of mycoplasma (MycoAlert™ Plus Mycoplasma Detection Kit, Lonza, USA) according to the manufacturer's recommendations.

### Generation of trastuzumab resistant SKTR subclone

SKBr-3 (SK) breast carcinoma cells were obtained from Eucellbank (University of Barcelona). SKBr-3 cells were routinely grown in McCoy's (Gibco) supplemented with 10% FBS (HyClone Laboratories), 1% L-glutamine, 1% sodium pyruvate, 100 U/mL penicillin, and 100 μg/mL streptomycin (Gibco). Trastuzumab-resistant SK cells (SKTR) were developed by exposing SK cells continuously to trastuzumab (Herceptin^®^, Hoffmann-La Roche Pharma), starting with 1μM concentration for three months of exposure and increasing the concentration up to 2 μM for a 12 months period, as we previously described [53]. Thus, cells resistant to trastuzumab were maintained in 2 μM trastuzumab, a concentration at which SKBr-3 parental cells were not viable (Figure 2).

### *In vitro* assay of trastuzumab sensitivity

The effect of trastuzumab on the growth of parental SKBr-3, trastuzumab resistant SKTR and JIMT1 cells was measured by the MTT reduction assay [54] according to the manufacturer's instructions (MTT Cell Proliferation Kit I, Roche). Cells were seeded at a density of 10^4^ cells/90μL medium/well in 96 well, flat bottomed tissue culture plates. Following overnight adherence, zero cell count was taken after which different concentrations of trastuzumab dissolved in sterile water were introduced to the wells. Control cells received vehicle without trastuzumab. Absorbance values were measured at 595 nm, with 690 nm as a reference in a multiplate reader (Thermo Scientific, Multiscan FC). Growth inhibition is expressed as the percentage of cell viability compared to untreated cells. IC_50_ value represents the trastuzumab concentration that caused 50% growth suppression. All measurements were done in at least six repeats.

### Gene expression measured by RT-qPCR

Cells were trypsinized and total RNA was extracted with the RNeasy kit (QA, Qiagen, Venlo, The Netherlands) according to the manufacturer's guidelines. 1 μg of total mRNA was reverse transcribed to cDNA (Maxima™ First Strand cDNA Synthesis Kit, Thermo Scientific). The expression of DUSP4 and DUSP6 along with HER2 was verified by LightCycler 480 DNA SybrGreen Master I (Roche) using Light Cycler 480 RT-qPCR instrument (Roche), each sample measured in triplicates. Gene expression values were normalized to an internal control gene (β-actin). NCBI Blast was used to eliminate potential cross-hybridization (http://blast.ncbi.nlm.nih.gov). Sequence of the used primers were: β-actin forward: 5’-CCCTGGAGAAGAGCTACGAG-3’, reverse: 3’-GAAGGAAGGCTGGAAGAGT-5’; DUSP6 forward: 5’-GCAGTTTCTCTTGGCAGCAT-3’, reverse: 3’-CGCACTTGGTAACCTTGTC-5’; DUSP4 forward: 5’-TGGAAGCCATAGAGTACATCGA-3’, reverse: 3’-CCTCACCCGTTTCTTCATCA-5’; HER2 forward: 5’-ACCTGGAACTCACCTACCTG-3’, reverse: 3’-ACTTGGTTGTGAGCGATGAG-5’.

### Gene silencing by siRNA

We employed siRNA concentration to achieve the highest silencing efficacy based on a previously tested GAPDH positive control siRNA (Silencer Select, Life Technologies) [55]. For both DUSP4 and DUSP6 two pre-designed Silencer Select siRNAs were tested (Silencer Select IDs DUSP4: s223450, s223451; DUSP6: s4379, s4380, Life Technologies), and the one with higher silencing efficacy was selected for subsequent drug combined silencing experiments. To provide a baseline to compare siRNA-treated samples, control cells received Negative Control siRNA No. 1 (Silencer Select, Life Technologies) that does not target any gene product. To perform silencing, 3×10^5^ cells/well were plated to each well of a 6 well plate in duplicates for each siRNA. Cells were transfected by Lipofectamine RNAiMax (Life Technologies) transfection reagent. After 24 hours, culture media was renewed and cells were incubated for an additional 48 hours. Cells were trypsinised and RNA silencing efficacy was measured by RT-qPCR. Expression of target genes from cells treated with Silencer Select siRNAs was compared to Negative Control siRNA transfected cells.

### RNA interference combined with trastuzumab treatment

To assess the role of selected candidate genes in trastuzumab resistance, we combined siRNA transfection with drug treatment on a 96 well plate. In each well, 10^4^ cells /90μL were plated out and transfected with 30 nm siRNA in six repeats. After overnight incubation, cell count was taken and 10 μg/ml/well trastuzumab was introduced, that also corresponds to the pharmacologically relevant concentration [56]. Control wells received 10 μL vehicle. After a 48 h drug treatment, MTT assay was performed and absorbance values were measured. The difference in viability between Negative control siRNA and target-gene transfected cells was calculated by Kruskall-Wallis test. Significance level was set at *p* < 0.05. A summary of the performed analysis steps is provided in Figure 1A.
